# Sex Allocation in California Oaks: Trade-Offs or Resource Tracking?

**DOI:** 10.1371/journal.pone.0043492

**Published:** 2012-08-27

**Authors:** Johannes M. H. Knops, Walter D. Koenig

**Affiliations:** 1 School of Biological Sciences, University of Nebraska, Lincoln, Nebraska, United States of America; 2 Lab of Ornithology and Department of Neurobiology and Behavior, Cornell University, Ithaca, New York, United States of America; Jyväskylä University, Finland

## Abstract

Trade-offs in sex resource allocation are commonly inferred from a negative correlation between male and female reproduction. We found that for three California oak species, aboveground annual net productivity (ANP) differences among individuals were primarily correlated with water availability and soil fertility. Reproductive biomass increased with ANP, but the relative allocation to reproduction was constant, indicating that reproduction tracked productivity, which in turn tracked site quality. Although there was a negative correlation between male and female reproduction, this was not the result of a resource investment trade-off, but rather a byproduct of the positive correlation between female reproductive biomass and ANP combined with the greater overall resource allocation to female, compared to male, function. Thus, we reject the hypothesis of a trade-off between these key life-history components within individuals of these species. For long-lived individuals, a plastic resource tracking response to environmental fluctuations may be more adaptive than directly linking life-history traits through trade-offs.

## Introduction

Trade-offs in resource investment between life history functions such as growth and reproduction, current and future reproduction, fecundity and longevity, and between male and female function are central themes of evolutionary biology [1], [2], [3]. Traits commonly thought of as exhibiting trade-offs in plants include: growth and reproduction, size and number of seeds, and, in the case of monoecious and hermaphroditic species, resource allocation to male and female function. The last, which determines the floral sex ratio in many plants, is known to vary considerably [4], [5], [6], [7], [8], [9] and has important consequences to fitness [1], [2], [10]. Empirical studies also have shown that males of dioecious plants often occur in more stressful environments, implying that either males require fewer resources than females or that the sexes have different patterns of resource allocation and that the optimal sex ratio may vary with environmental conditions [1], [6], [7], [8], [9], [11], [12]. Modeling also shows that the evolution of habitat-dependent sex allocation may be favored, with because female reproduction is requires more resources, increased female reproduction in fertile and increased male reproduction in infertile patches if pollen disperses more widely than seeds [13].

Four hypotheses have been proposed potentially explaining increased fecundity caused by differences in allocation to male and female function and how this variable sex allocation is habitat-dependent among wind pollinated monoecious plants. First, given the assumptions that female reproduction in flowering plants requires a larger investment and that larger individuals are presumed to have, in general, more resources available, there should be increased female allocation with size [14], [15], [16]. However, this hypothesis has been questioned for wind pollinated species, where often increased male allocation with size has been observed [17], [18], [19]. Second, large, tall individuals may have increased male allocation because of more effective pollen dispersal [20]. Third, large individuals may have increased male allocation, because wind dispersed pollen does not saturate, whereas female allocation is expected to be a decreasing function of seed number because of limited dispersal leading to stronger resource competition among seedlings [13], [21]. Fourth, increased male allocation with size might be caused by a constraint in plant architecture, if male and female flowers are arranged differently and branching patterns change with size [14], [18], [19].

The male height advantage and the pollen saturation hypotheses predict that large, tall individuals should have increased male allocation because of increased fitness gain caused by higher resource allocation to male function, a finding supported by some experimental studies [22]. Alternatively, Masaka and Takada [23] developed a model of floral sex ratio in wind-pollinated monoecious plants that combined wind pollination efficiency with competitive sharing among male flowers. Because wind pollination is inefficient, pollination efficiency in this model follows a Poisson rather than a normal distribution, and denser populations have increased pollination efficiency that should favor increased allocation to male function, a benefit expected to be largest for the smallest individuals. This model predicts that above a size threshold, male allocation does not vary with resource availability, whereas female allocation increases with size [24].

A more general model by Zhang and Jiang [25] also concludes that above a size threshold a fixed proportion of resources should be allocated to male function, assuming that the female fitness gain is a linear function of resource investment. In their model, male and female allocations are predicted to be positively correlated if both male and female functions exhibit a saturating function with resource investment.

Field studies generally examine sex allocation by comparing individuals that vary in resource availability [7], [11], [12]. Observed differences in reproductive allocation may therefore be a consequence of male or female reproduction varying with resource availability rather than with each other [25]. If either male or female reproductive effort is plastic and tracks resource availability, the resulting sex allocation would potentially show a negative correlation, but this would not imply a phenotypic trade-off [26], [27], [28], [29]. Such “sexual resource tracking” provides an alternative way for individuals to optimize their performance depending on the environment and available resources, without requiring any genetic differences in sex allocation among individuals [30]. Sexual resource tracking is particularly likely to be applicable to long-lived individuals in environments that vary in resources. For such species the timing of resource investment often differs between male and female function and thus seasonal differences in resource availability are likely to influence male and female function in different ways [31].

Resources are also likely to vary over the lifetime of an individual, especially for long-lived trees. For example, if water is limiting for growth and reproduction, water availability will increase over time because deeper soil water becomes more usable as the rooting system develops. Plasticity in life history factors, rather than trade-offs, is likely to be a more efficient strategy to optimize use of temporal and spatial variability in resources.

To test these resource allocation models and evaluate sexual allocation differences among habitats, it is necessary to quantify not only male and female reproductive effort but also vegetative investment allowing the evaluation of how male and female reproductive effort scales with total resource availability. Unfortunately, such data are largely missing in the literature because reproductive studies focus primarily on reproduction while ecosystem productivity studies typically do not separate male and female reproductive effort.

We examined reproductive biomass and allocation, annual aboveground net productivity (ANP), and site quality for 40 individuals of three monoecious oak species (genus *Quercus*) over a five-year period using litterfall data. Each of the three oak species exhibited large variability in annual and individual seed production [32]. We first examined if reproduction tracked resource availability or if there was trade-off in resource allocation which predicts a negative relationship between total reproductive and vegetative growth that changed along a gradient of resource availability. Second, we examined if there was a trade-off between male and female reproduction, or if male and female biomass each tracked resource availability independently. Third, we tested for the possibility that female reproductive effort increased continuously with increased resource availability while allocation to male reproductive effort does not increase with increased resource availability as a means of preventing mate competition as has been suggested for wind pollinated species [23], [24], [33].

## Results

### ANP

**Figure 1:**
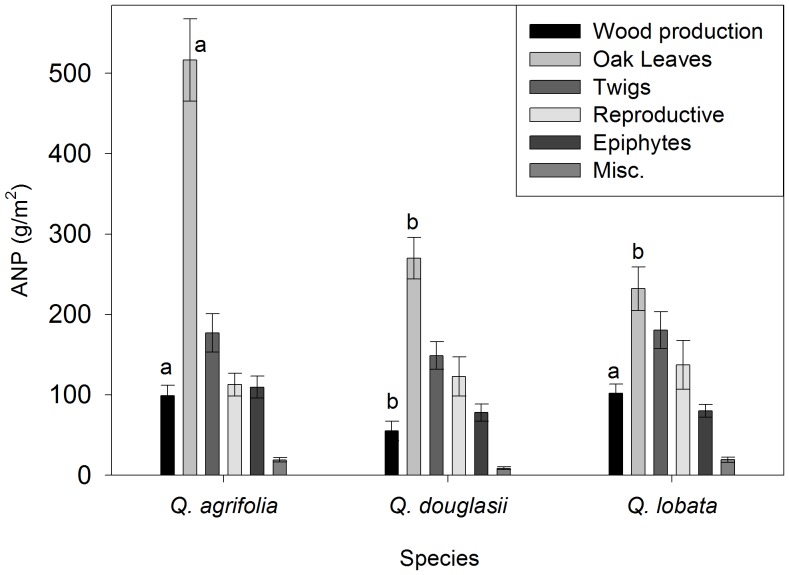
Five-year average (± SE) ANP of *Quercus agrifolia* (n = 13), *Q. douglasii* (n = 13 and *Q. lobata* (n = 14). Reproductive litterfall consisted of acorns, acorn caps, aborted acorns (including unfertilized flowers) and catkins. Epiphytes included lichens, mosses and mistletoe. Miscellaneous includes insects, insect frass, and unidentifiable organic material. Significant differences among species include oak leaves (one-way ANOVA, *F*_2,37_ = 18.1, *P*<0.001) and woody increment (*F*_2,37_ = 5.5, *P*<0.01). Different letters denote *P*<0.05 in a Tukey post-hoc comparison among species.

The five-year average ANP of *Q. agrifolia* was 43% higher than *Q. douglasii* and *Q. lobata* (one-way ANOVA, R^2^ = 0.23, *P*<0.01), caused primarily by significantly higher leaf production (Fig. 1). The only other ANP category differing among species was estimated woody growth, with *Q. douglasii* being significantly lower than the other two species.

**Figure 2:**
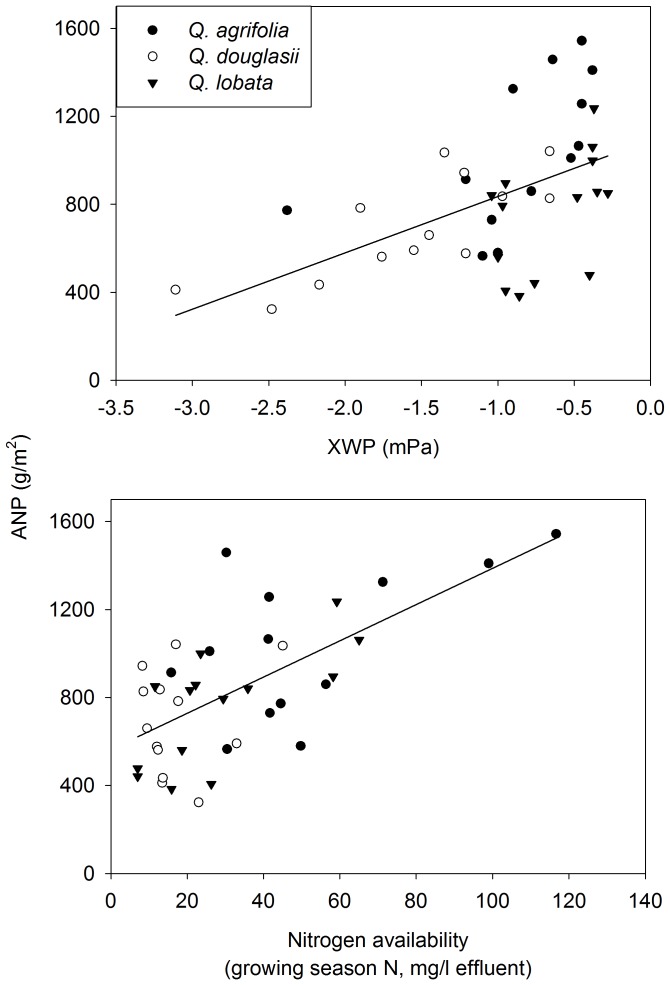
Five-year average (± SE) ANP of *Quercus agrifolia* (n = 13), *Q. douglasii* (n = 13 and *Q. lobata* (n = 14) in relation to XWP and resin bag extracted nitrogen. Lines are drawn for statistically significant relationships based on the GLM (Table 1).

Within all three species, ANP varied 3-fold (Fig. 2). However, independent of species identity, ANP differences among individuals strongly and linearly correlated with site quality as determined by water and nitrogen availability (Table 1, Fig. 2). Thus productivity differences among individuals and species reflected differences in site quality.

**Table 1:** GLM of aboveground annual net productivity (ANP), with as independent factor species, and as covariates pre-dawn xylem water potential (XWP) and soil mineral nitrogen (resin N).

Variable	df	*F*-value	*P*-value
Model	4,33	8.7	0.000
Species	2,33	1.0	0.400
XWP	1,33	18.3	0.000
Resin N	1,33	9.7	0.004
Species * XWP	1,33	0.4	0.698
Species * Resin N	1,33	1.1	0.362

*N* = 13 (*Q. agrifolia* and *Q. douglasii*) and 14 (*Q. lobata*). ANP is the averaged five-year litterfall and wood increment for each tree. Overall *R* ^2^ = 0.69.

### Reproductive Biomass

**Figure 3:**
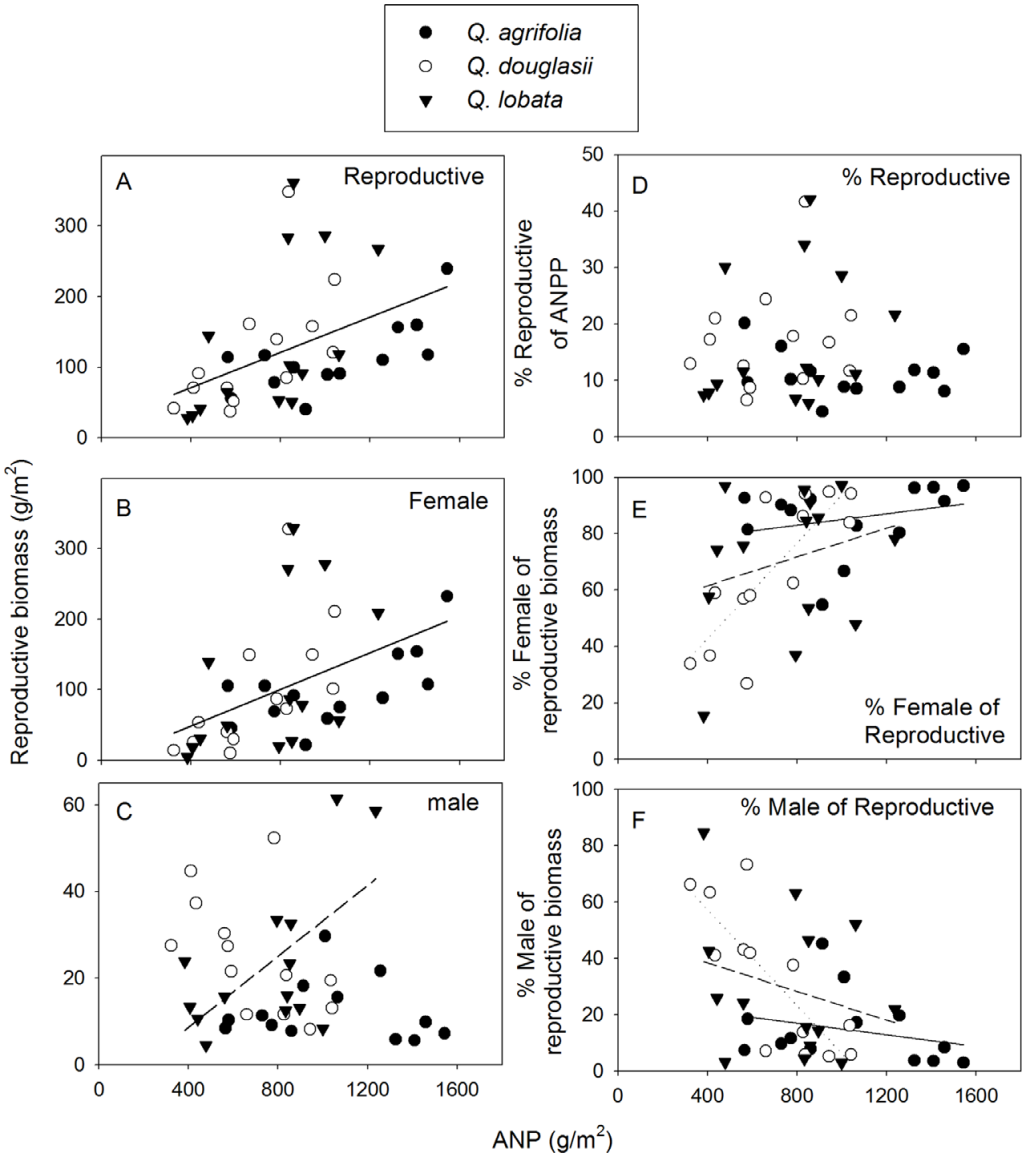
Annual aboveground net productivity (ANP) in three California oak species in relation to reproduction. Shown are ANP in relation to (A) total reproductive biomass, (B) female reproductive biomass, (C) male reproductive biomass, (D) percent reproductive biomass of ANP, (E) percent female reproductive biomass of total reproduction, and (F) percent male reproductive biomass of total reproduction. Plotted are the five year means of the 40 individual trees. ANP was calculated from aboveground litterfall and an estimate of trunk increment based on dendrometers. Lines are drawn for species averages in A and B, *Q. lobata* only in C, and in E and F solid line for *Q. agrifolia*, long dashed line *Q. lobata* and short dashed line *Q. douglasii* based on GLMs (Table 2). For *Q. lobata* only there was a significant positive relationship between ANP and male allocation (*R* ^2^ = 0.38, *P*<0.02).

There were no significant differences among species in the proportion of ANP allocated to reproduction (one-way ANOVA, *P*>0.7, Fig. 1.). However, reproductive biomass varied much more than ANP within species, from 6-fold in *Q. agrifolia* to 13-fold in *Q. lobata*, and was as high as 42% of total ANP (Fig. 3a). Total reproductive biomass was positively correlated with total ANP (Fig. 3a) but there were no significant differences among species (Table 2), nor did the relative allocation to reproduction as a proportion of total biomass vary with ANP (Fig. 3d, Table 2). We also found that nitrogen and phosphorus had the same relationship to reproduction as biomass (Fig. S1).

**Table 2:** GLMs of reproductive biomass, % reproductive biomass of ANP, female and male reproductive biomass, and % of male/female allocation as a % of reproductive biomass, with as independent factors species and as a covariate, aboveground annual net productivity (ANP).

Factor _df_	df	Reproductive		% Reproductive		Female		Male		% Male/Female	
R^2^		0.46		0.15		0.45		0.37		0.41	
		F	P	F	P	F	P	F	P	F	P
Model	5, 34	5.7	0.001	1.2	0.330	5.6	0.001	4.0	0.006	4.7	0.002
Species	2,34	0.8	0.467	0.5	0.616	1.1	0.335	4.8	0.015	2.8	0.076
ANP	1,34	24.7	0.000	0.4	0.547	22.0	0.000	0.0	0.899	10.7	0.003
Species * ANP	2,34	0.9	0.419	0.6	0.547	1.2	0.306	4.7	0.016	2.7	0.081

*N* = 13 (*Q. agrifolia* and *Q. douglasii*) and *N* = 14 (*Q. lobata*). ANP and reproductive litterfall are five-year averages for individual trees.

### Female and Male Reproduction

**Figure 4:**
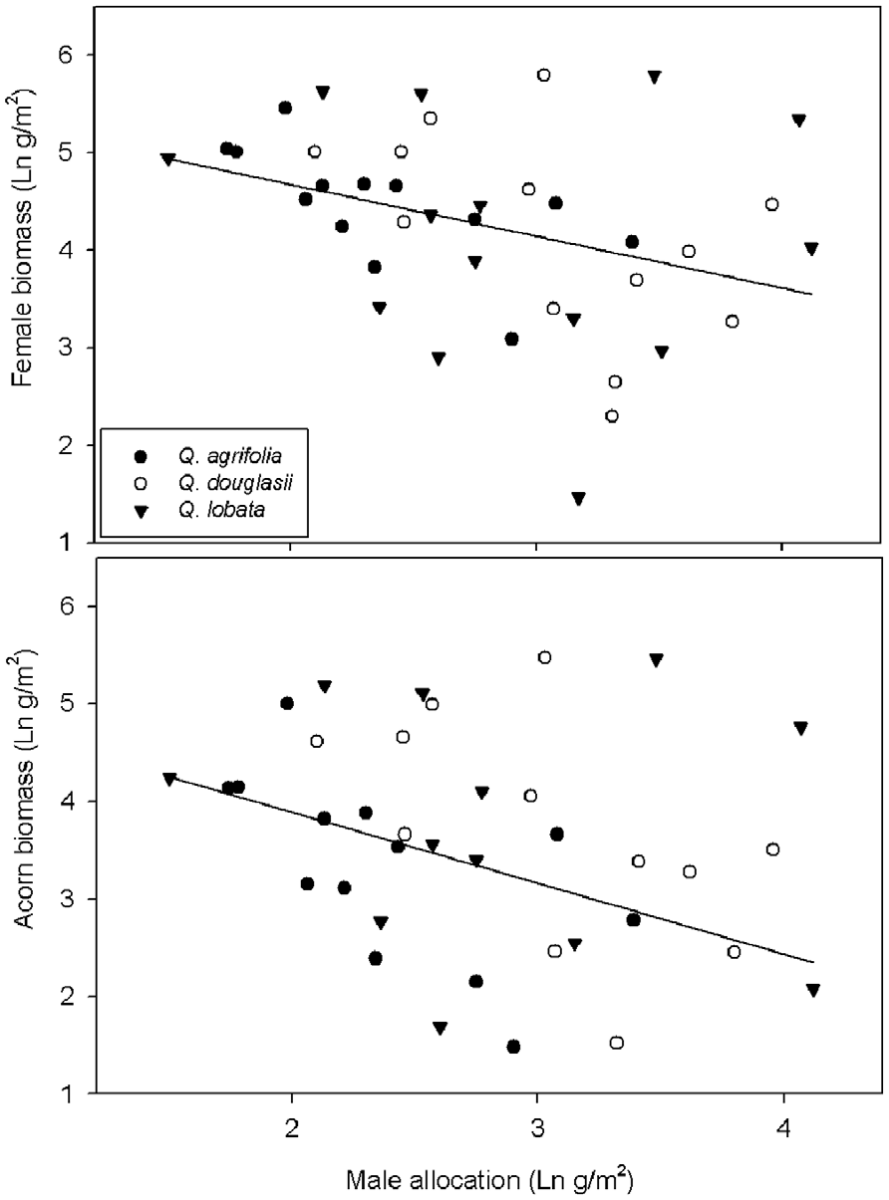
Five-year average (± SE) male reproductive biomass of 40 trees of *Quercus agrifolia, Q. douglasii* and *Q. lobata* in relation to female reproductive biomass. Shown are (A) female reproductive biomass (acorns, acorn caps and aborted acorns, including unfertilized female flowers) (Regression, *n* = 40, *F* = 5.3, *P*<0.05, R^2^ = 0.35) and (B) female acorn biomass (Regression *n* = 40, *P*<0.05, R^2^ = 0.33). Species were not significantly different (all *P*>0.4).

Male reproductive biomass was negatively correlated with both acorn productivity and total female reproductive biomass (Fig. 4). Female allocation closely matched total reproductive allocation (Fig. 3a, b), a result that was not surprising given that 84% of total reproductive biomass was female. Female reproductive biomass also showed a positive relationship with total ANP (Fig. 3b) and there were no significant differences among species in this relationship (Table 2). Female reproductive biomass varied much more than total reproduction within species, varying over a factor of 11 for *Q. agrifolia*, 33 for *Q. douglasii* and 75 for *Q. lobata* (Fig. 3b). In terms of the components of female reproductive biomass, acorn caps contributed 28%, acorns 55%, and aborted acorns 17%. Acorn caps, acorns and aborted acorns closely matched each other and total female reproductive biomass (data not shown). Thus, as for total reproductive effort, female reproductive biomass increased with increased productivity and there were no significant differences among species.

Female allocation expressed as a percent of total reproductive effort plotted against ANP exhibited a pattern similar to female reproductive effort. However, species differed significantly in this relationship with *Q. douglasii* having the strongest and *Q. agrifolia* having the weakest relationship (Table 2, Fig. 3b, e).

On average, trees invested less in male reproductive biomass than female reproductive biomass (Fig. 3b, c). Furthermore, female investment was much more variable; individual trees varied 5 (*Q. agrifolia*) to 14 times (*Q. lobata*) more in female, as compared to male reproductive effort (Fig. 3c). In contrast to female reproduction, there were significant differences in male reproductive biomass among species with *Q. agrifolia* allocating only half as much to male reproduction compared to *Q. douglasii* and *Q. lobata* (one-way ANOVA, R^2^ = 0.15, *P*<0.05). Male reproductive biomass did not increase with ANP for *Q. agrifolia* and *Q. douglasii*, however, it was significantly positively correlated with ANP for *Q. lobata* (R^2^ = 0.38, *P*<0.02). Male reproductive allocation was not significantly affected by the tree height (data not shown).

Male plus female reproductive allocation sums to 100% for each individual tree. Consequently, these variables show the same opposite relationship with ANP as female reproductive allocation (Fig. 3d, f).

### Acorn Numbers

The total number of initiated and aborted acorns (including unfertilized flowers) correlated positively and significantly with ANP. Filled acorns showed the same relationship, although it was not significant (Table S1, Fig. S2). There was no significant relationship between the proportion of filled acorns and ANP (Table S1). Thus, trees in productive sites initiated more acorns and the pattern of increased female reproductive effort in more fertile sites was not the result of selective abortion of acorns in unproductive sites.

## Discussion

Evidence presented here indicates that ANP differences among individuals of the three California oak species studied reflect differences in site quality, and that differences in reproductive biomass reflect differences in ANP. These findings support the hypothesis that both productivity and reproductive investment track resource availability rather than that there is a trade-off between growth and reproduction along a resource gradient, a result consistent with a previous study that examined reproduction and woody growth of these species [34]. Thus, similarly to Hulshof et al [35] who found for a dioecious tropical tree a different allometric scaling of growth and reproduction with resource availability, we found for reproduction different allometric scaling of male and female reproduction with resource availability. We also found significant plasticity in female reproductive biomass with more female flowers maturing at higher ANP, a result most likely a direct consequence of higher resource availability. Male reproduction showed less variation and only one species had higher absolute (but not relative) male reproduction in relation to ANP.

Our finding that female allocation increased with increasing resource availability, combined with a relatively fixed male allocation, matches the prediction of Masaka and Takada [23], based on a model combining wind pollination efficiency with competitive sharing among male flowers. This model combines individual resource limitation with pollen limitation to predict temporal patterns of masting [36], [37]. Clearly low pollination efficiency and pollen dispersal are key aspects driving the ecology and evolution of wind-pollinated tree species [38], [39], [40].

The mechanism behind this pattern can be clarified by considering male and female reproduction separately. Female reproductive biomass of all three species was positively correlated with total ANP, whereas male reproduction was not in two of the species. A similar positive correlation between female reproduction and ANP has been n found for Ponderosa pine [41] and for a perennial herb [16].

A potential cause of male/female differences in allocation may be the timing of the investment in male and female reproduction. Male catkin production occurs over a short period in the early spring when the environment in this Mediterranean climate is still typically wet and when differences in site quality due to water are minimal. As a result, resources available for male reproduction are probably relatively high, regardless of overall ANP, and there is consequently no relationship between absolute male reproductive biomass and resources. In contrast, female flowers, although initiated in the spring, develop over a much longer time period, and the greater part of female investment occurs during the summer when there are large differences among individuals in water availability and site quality [42], [43] allowing individuals in wetter, more fertile sites to photosynthesize more and invest more in maturing seeds during the summer. Thus female investment increases with resource availability and male investment does not. Other studies have shown that nitrogen or phosphorus rather than biomass can be the key limiting resource for plants [44], [45], [46], [47]. However, we found that nitrogen and phosphorus exhibit the same allocation pattern as biomass (Fig. S2). We also did all allocation calculations and analysis with nitrogen and phosphorus instead of biomass and found the same patterns (data not shown).

Most of the literature examining sex allocation in relation to plant size [48], including the few wind pollinated tree species examined [24], [41], [49] have found increased female allocation with increased size [16], [21], consistent with our result of increased female allocation with increasing ANP. In wind pollinated herb species, however, such studies have generally found the opposite pattern of increased male allocation with size [20], [22], [50]. This difference may be related to pollen limitation as it is affected by plant size. Specifically, it is likely that a relative increase in height for short-stature, short-lived plants may increase pollination much more than for tall trees.

A necessary condition for a trade-off in sex allocation is a negative correlation between the relative allocation of total reproduction to male and female function. Although this was the case in the oaks we studied, a negative correlation is necessary but not sufficient to demonstrate a trade-off. In this case, no trade-off appears to exist; rather, the negative correlation is a byproduct of female reproductive biomass that strongly tracks available resources in all three species, whereas male reproduction is relatively fixed. As pointed out by Zhang and Jiang [25], it is critical to not only consider gender plasticity, but also absolute measures of male and female reproduction because total reproductive effort potentially confounds relative sex allocation.

Trade-offs are a key element in life history evolution. The strongest evidence of sex allocation trade-offs comes from short-lived organisms, where individual fitness differences can be closely linked to life-history differences [4], [5], [8], [51]. In contrast, our findings focus on long-lived trees that can reproduce over hundreds of years. We found no evidence for a trade-off between male and female sex allocation for three long-lived oak species that exhibit highly variable and synchronized reproduction (i.e. “masting” behavior).

Previously we have presented evidence in California oaks for a lack of a direct trade-off between growth and reproduction [34], and between seed size and number [52]. Across years, however, individuals in this population exhibited some apparent trade-offs, although patterns were variable and sometimes varied with respect to resources in unexpected ways [53]. For example, Barringer et al. (Oecologia, in press) found more of an apparent trade-off between acorn production in years when resources were relatively abundant than when they were scarce, contrary to expectations. In any case, for long-lived individuals, a plastic resource tracking response to environment fluctuations may be a more adaptive response than directly linking life-history traits through trade-offs. Thus evolutionary changes are likely to be not only slower [54], but might also be much less pronounced in long-lived species. Such plasticity is likely to be combined with trade-offs among years, as suggested by strong negative temporal autocorrelations in acorn production [32], [34], [40].

Long-lived species such as the oaks studied here potentially exhibit strikingly different patterns of life-history variation than short-lived species despite correlations among life-history characters superficially suggestive of similar kinds of trade-offs. Based on the results presented here, no evolutionary trade-off need be invoked to explain patterns of reproductive allocation in these species of California oaks. Rather, the negative correlations between male/female resource allocation among individuals can be explained by resource tracking.

## Methods

We have been conducting a long-term seed production study, which include 87 *Quercus lobata* (valley oak), 57 *Quercus douglasii* (blue oak), and 63 *Q. agrifolia* (coast live oak) individuals At Hastings Reservation in central, coastal California since 1980 [32]. Based on the average seed production from 1980–1990 we divided the individuals within each species in three groups, low, medium and high seed producers, and stratified with each group, randomly selected 13 *Q. agrifolia*, 13 *Q. douglasii*, and 14 *Q. lobata* individuals. *Q. agrifolia* is evergreen, while both *Q. douglasii* and *Q. lobata* are deciduous. *Quercus* species are wind pollinated, largely self-incompatible [55], and rates of pollen flow are high [56], [57]. All three species produce a new flush of growth each spring, with male catkins at the base of the new growth and female flowers in the axil of the leaves. Acorns mature during the summer and fall in the same year as pollination for all three species. Data was collected during several years of high acorn production (“mast” years) and for all three species the average acorn crop over the study period was close to the 30-year average (1980–2010, J. Knops & W. Koenig, unpublished data).

We collected litterfall monthly from November 1991 through March 1997. We measured the canopy projection of each individual tree and placed litter collectors at randomly-selected spots under the canopy. Woody increment was converted to an area basis based on the measured canopy projection area for each tree. All values are expressed as m^2^ canopy area, which adjusts for tree size differences. Litterfall was collected in black plastic plant pots with a top diameter of 50 cm and an area of 0.196 m^2^. Collectors were 40 cm high and contained 6 holes in the bottom that were covered with pieces of fish netting to allow rainwater passage. Litterfall sampling consisted of three buckets per tree. A small proportion of buckets tipped over (196 [2.5%] out of 7,800 samples). When this occurred, we used the average litterfall of the two remaining buckets. There were also 22 cases where all three buckets were lost in a single month (0.9% out of a total of 2,602 tree-months). In these cases, we used the species average for the month in question to estimate each missing individual’s average. All trees were spatially separated by at least 50 m but were all within 3.5 km of each other and at least an estimated 50 years old.

Litter was sorted into oak leaves, twigs and branches, acorns, acorn caps, aborted acorns (including unfertilized flowers), catkins, epiphytes and miscellaneous. We averaged the three collectors within each individual each month and then totaled those averages to get an average for each individual tree for each year for the period from 1 March through the end of February. We included wood increment and litter from foliage, twigs, and reproductive structures in our estimate of ANP. All mature and aborted acorns (including unfertilized flowers) were also counted in 1992 and 1993.

Wood increment was measured in two ways. First, we placed dendrometers [58] at breast height around the trunk of all trees in the summer of 1994 and averaged the annual tree trunk increment from 1995 to 1999. Secondly, we directly measured tree-ring increment from *Q. douglasii* and *Q. lobata* cores and averaged the annual growth from 1990 to 1994; tree-rings of *Q. agrifolia*, an evergreen species, were too indistinct to measure. These two measurements of wood increment were linearly correlated (regression, ß = 1.01, *R* ^2^ = 0.84, *F*_1,25_ = 128, *P*<0.001). Thus, for the analysis presented here we used only the dendrometer measurements because they provided data for all three species. We estimated tree woody biomass based on tree volume equations for all three species provided by Pillsbury and Stephens [59]. Based on the annual trunk volume increment, we estimated the total woody mass increment assuming that green wood biomass contains 20% water (J. Knops unpublished data).

For total nitrogen and phosphorus analyses, leaf samples were pooled by tree and years. All other litter categories were pooled by species and across years. Samples were oven dried at 60°C and ground using a #10 screen in a Wiley Mill. Samples were digested using the standard Kjeldahl digestion method in sulfuric and salicylic acid [60], [61], [62] and analyzed on a continuous flow Technicon autoanalyzer.

Species are intermixed at the study sites. However, *Q. lobata* tends to occur on alluvial sites with groundwater, *Q. douglasii* on dry south facing slopes, and *Q. agrifolia* along seasonal streams and on relatively wetter north facing slopes. Site differences in water availability were estimated by measuring predawn xylem water potential (XWP) during September (the driest period of the year in this Mediterranean climate) in 1991, and again in each year from 1994 through 1998 [42], [43]. XWP differences among trees were highly concordant among years [43] and thus we used data only from 1991. Soil nitrogen availability was estimated with in situ resin bag accumulated ammonium and nitrate from mid-October 1992 through mid-April 1993; for details see Knops and Koenig [63]. This period captures the wet season, during which nitrogen mineralization occurs [64].

All statistics were performed with SPSS 19 for Windows. All data were averaged across the five years within each individual tree, except for the XWP and the nitrogen mineralization, which were only collected in one year. Among species differences were compared with one-way ANOVAs, and data was log transformed for normality when needed. Regressions were used to determine male versus female allocation, and reproductive allocation in relation to ANP was analyzed by General Linear Models (GLM) with least significant difference (LSD) post-hoc comparisons to test for differences among species. We also examine reproductive allocation with Generalized Linear Models, which in several cases fit better according to the Akaike Information Criterion (AIC). However, this did not change any of the significances found with the GLM analyses.

## Supporting Information

Figure S1 **Biomass investment as a percent of total ANP in reproductive structures, versus nitrogen and phosphorus investment in reproductive structures.**
(TIF)

Figure S2 **Total, aborted (included unfertilized flowers) and mature filled acorns for three California oak species in relation to the total annual aboveground net primary productivity (ANP).**
(TIF)

Table S1 **GLM of total, aborted (including unfertilized flowers), filled mature acorns and the percent of total acorns filled, with as independent factor species and ANP as a covariate.**
(DOCX)
