# Supplementary figures and images for: Sex Allocation in California Oaks: Trade-Offs or Resource Tracking?
Source: PLoS One. 2012 Aug 27;7(8):e43492. doi: 10.1371/journal.pone.0043492 (PMC3428368; doi:10.1371/journal.pone.0043492)

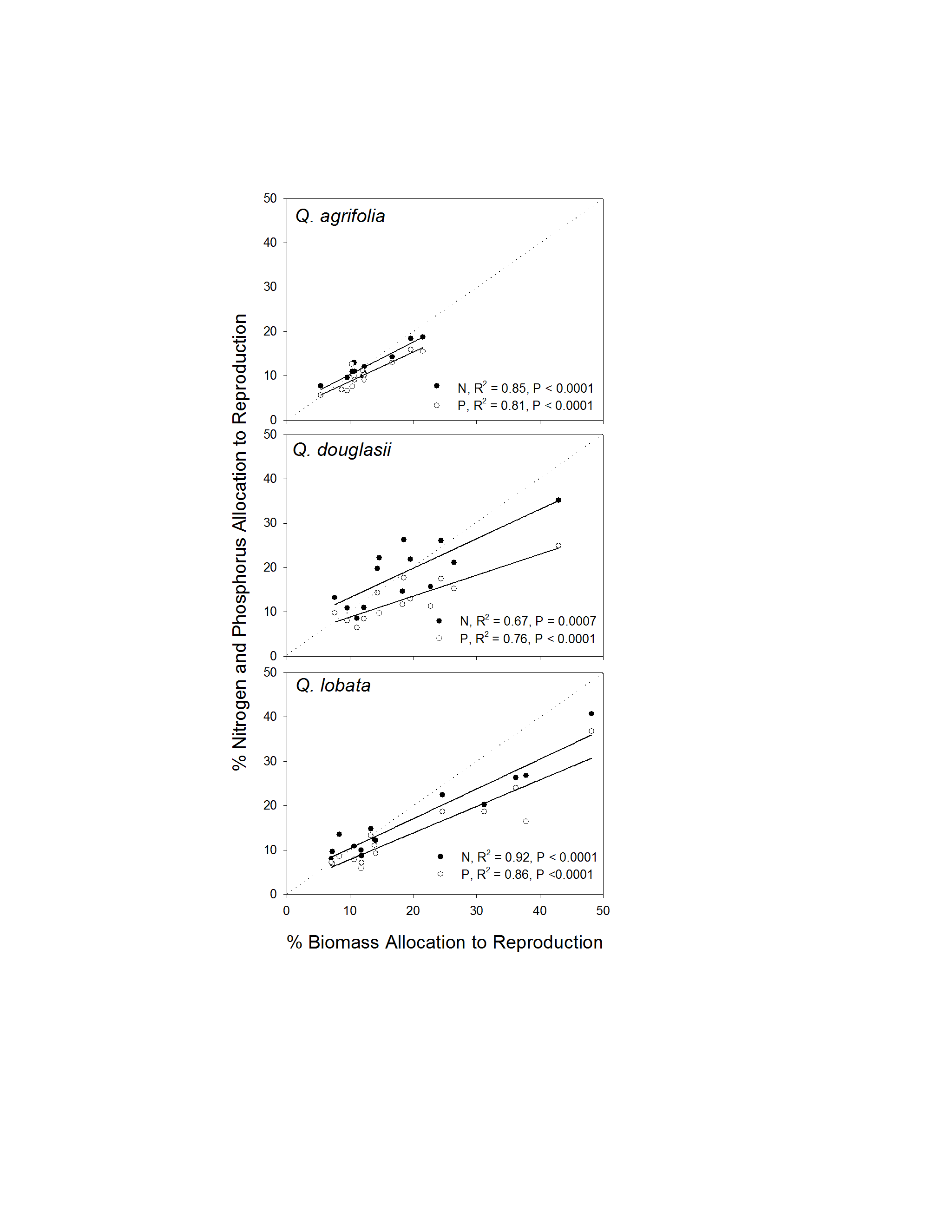

Supplement: Figure S1 — Biomass investment as a percent of total ANP in reproductive structures, versus nitrogen and phosphorus investment in reproductive structures. (TIF) [file pone.0043492.s001.tif]

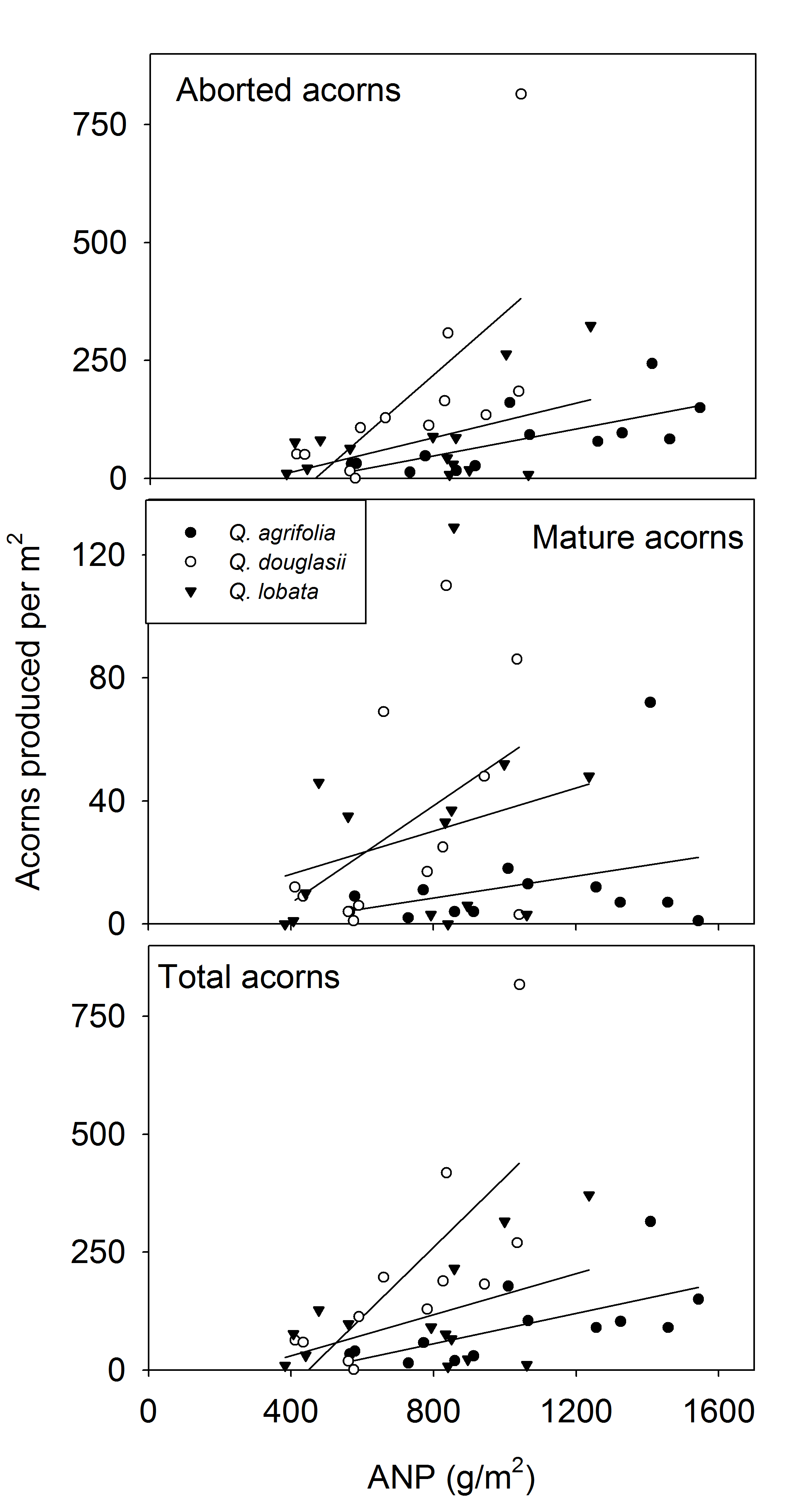

Supplement: Figure S2 — Total, aborted (included unfertilized flowers) and mature filled acorns for three California oak species in relation to the total annual aboveground net primary productivity (ANP). (TIF) [file pone.0043492.s002.tif]
