# Supplementary material for: Sex Allocation in California Oaks: Trade-Offs or Resource Tracking?
Source: PLoS One. 2012 Aug 27;7(8):e43492. doi: 10.1371/journal.pone.0043492 (PMC3428368; doi:10.1371/journal.pone.0043492)
Supplement: Table S1 — GLM of total, aborted (including unfertilized flowers), filled mature acorns and the percent of total acorns filled, with as independent factor species and ANP as a covariate. (DOCX) [file pone.0043492.s003.docx]

Table S1. GLM of total, aborted (including unfertilized flowers), filled mature acorns and the percent of total acorns filled, with as independent factor species and ANP as a covariate. (*n* = 13 for *Q. agrifolia* and *Q. douglasii*; n = 14 for *Q. lobata*). Acorns are the sum of 1992 and 1993 and ANP is a five-year average. Given are the F and P values of a GLM with species as a factor and the productivity as a covariable.

| Factor _df_ | Total | | Aborted | | Filled | | Filled as % of total | |
| --- | --- | --- | --- | --- | --- | --- | --- | --- |
| R^2^ | 0.48 | | 0.45 | | 0.17 | | 0.419 | |
|  | F | P | F | P | F | P | F | P |
| Model _5, 33_ | 6.1 | 0.000 | 5.3 | 0.001 | 1.4 | 0.256 | 0.9 | 0.509 |
| Species _2, 33_ | 1.9 | 0.176 | 2.7 | 0.083 | 0.1 | 0.730 | 3.9 | 0.057 |
| ANP _1,33_ | 20.9 | 0.000 | 17.2 | 0.000 | 5.4 | 0.027 | 0.4 | 0.689 |
| Species * ANP _2, 33_ | 5.8 | 0.007 | 0.7 | 0.009 | 0.3 | 0.716 | 0.4 | 0.654 |
|  |  |  |  |  |  |  |  |  |
